# Distinct regional ontogeny and activation of tumor associated macrophages in human glioblastoma

**DOI:** 10.1038/s41598-020-76657-3

**Published:** 2020-11-11

**Authors:** Alexander P. Landry, Michael Balas, Saira Alli, Julian Spears, Zsolt Zador

**Affiliations:** Division of Neurosurgery, Department of Surgery, St. Michael’s Hospital, Toronto, ON Canada

**Keywords:** Cancer microenvironment, CNS cancer, Tumour heterogeneity, Tumour immunology, Functional clustering, Gene regulatory networks, Machine learning

## Abstract

Tumor-associated macrophages (TAMs) constitute up to 50% of tumor bulk in glioblastoma (GBM) and play an important role in tumor maintenance and progression. The recently discovered differences between invading tumour periphery and hypoxic tumor core implies that macrophage biology is also distinct by location. This may provide further insight into the observed treatment resistance to immune modulation. We hypothesize that macrophage activation occurs through processes that are distinct in tumor periphery versus core. We therefore investigated regional differences in TAM recruitment and evolution in GBM by combining open source single cell and bulk gene expression data. We used single cell gene expression data from 4 glioblastomas (total of 3589 cells) and 122 total bulk samples obtained from 10 different patients. Cell identity, ontogeny (bone-marrow derived macrophages-BMDM vs microglia), and macrophage activation state were inferred using verified gene expression signatures. We captured the spectrum of immune states using cell trajectory analysis with pseudotime ordering. In keeping with previous studies, TAMs carrying BMDM identity were more abundant in tumor bulk while microglia-derived TAMs dominated the tumor periphery across all macrophage activation states including pre-activation. We note that core TAMs evolve towards a pro-inflammatory state and identify a subpopulation of cells based on a gene program exhibiting strong, opposing correlation with Programmed cell Death-1 (PD-1) signaling, which may correlate to their response to PD-1 inhibition. By contrast, peripheral TAMs evolve towards anti-inflammatory phenotype and contains a population of cells strongly associated with NFkB signaling. Our preliminary analysis suggests important regional differences in TAMs with regard to recruitment and evolution. We identify regionally distinct and potentially actionable cell subpopulations and advocate the need for a multi-targeted approach to GBM therapeutics.

## Introduction

Glioblastoma (GBM) is the most frequent adult primary brain tumor which remains invariably lethal. Median survival is approximately 16–20 months^1^ and has remained essentially unchanged over the last decades^2^. It is known that this tumour’s ability to reprogram the body’s immune response in order to evade destruction and support growth is one of the key mechanisms of glioblastoma resilience^3,4^. Most of the immune cells in GBM consist of tumor associated macrophages (TAM), which may constitute up to 50% of tumor bulk^5^. TAMs may be recruited from circulating bone marrow derived macrophages (BMDM) or resident microglia^3,6^. Notably, recent studies have implied distinct regional abundance for TAMs, with BMDM being dominant in tumor core while microglia are more prevalent in the invading edge^7^. TAMs are also described in terms of their activation state, often categorized as “classical” (M1) with seemingly anti-tumor effects or “alternative” (M2) with pro-tumor effects, both arising from a precursor “pre-activation” state (M0)^8^. However, emerging evidence suggests that TAM activation in-vivo doesn’t translate well to this categorical model^9^. Therefore, recent approaches use a score-based method that to assess macrophage activation states^10,11^. Understanding the origin of TAMs and the process by which their oncotoxic functions are silenced may provide the basis for new treatment options. Notably, immune checkpoint blockade has been advocated as one of the key modulators of T cell activation based on its game-changing effects in melanoma^12^, non-small cell lung cancer^13^ and Hodgkin’s lymphoma^14^. However, PD1 inhibitors have shown prolonged benefit for only a small patient group (< 10%) within a GBM cohort^15^. Furthermore, PD-1 expression has been recently demonstrated in TAMs with anti-phagocytic effects^16^ implying that PD-1 may exert its effect through mechanisms other than modulating T-cells.

Inter- and intra-tumoral heterogeneity of the cellular environment in GBM is well documented^7,17–19^. Regional differences are particularly distinct between the hypoxic tumor core and the invading edge which interfaces with adjacent brain tissue^20–22^. Recent data further supports the relevance of GBM diversity: features such as neoplastic cell composition^23^, mutation load^24^, and immune cell composition^25^ associate with GBM treatment response to radiation therapy, chemotherapy, and even immune modulation. Here we set out to further explore the heterogeneity of glioblastoma microenvironment using established techniques in computational biology. We hypothesize distinct region-specific recruitment, activation, and maturation programs.

## Methods

### Data pre-processing

The majority of our analysis relies on single cell RNA-sequencing (scRNA-seq) data obtained by Darmanis et al^7^, made publicly available on Gene Expression Omnibus (GEO)^26^ accession GSE84465. This dataset includes 3589 cells from 4 IDH wild type glioblastomas, each with paired samples from the tumour periphery (outside the region of gadolinium enhancement on MRI) and tumour core (the region of enhancement on MRI). The authors used antibody-mediated cell sorting, RNA cluster-based sorting, and copy-number variation analysis to identify seven cell types: immune, oligodendrocytes, oligodendrocyte precursors (OPCs), vascular, neurons, astrocytes, and neoplastic cells. Importantly, the original paper characterizes immune cells based on myeloid-specific genes and find that > 95% of immune cells are represented by macrophages or microglia. Therefore, lymphoid cells are not considered in this paper and immune cell are taken to be macrophages. It is known that GBM is relatively lymphodepleted compared to other cancers, making this approximation more feasible^27^. Full details of tumour collection, RNA-sequencing, and quality control parameters can be found in the original paper. Spike-in quality control genes were omitted from our analysis. The package Seurat^28^ (version 3.1.4) was used to preprocess the data. Firstly, the tumour label was regressed out of the raw counts and the resulting data was scaled and centered. Counts from each cell were divided by the cell’s total counts, multiplied by a default scale factor (10,000), and log-transformed. This processed data, as well as the raw count data, was used in all subsequent analysis.

To compare peritumoral microglia to normal microglia, we obtained publicly available scRNA-seq data on homeostatic microglia from GEO accession GSE135437 (960 cells from five human samples)^29^ and GSE134705 (2,304 cells from six human samples)^30^. The aforementioned preprocessing steps were used on both datasets. After aggregating the homeostatic and peritumoral microglia datasets together, we performed Weighted Gene Coexpression Network Analysis (WGCNA) to identify gene modules that are distinct to either homeostatic or peritumoral microglia^31^.

In order to perform validation of our findings we relied on publicly available bulk RNA-seq data from the Ivy Glioblastoma Atlas Project^22^, using a subset of of 122 RNA samples which were obtained from 10 different tumours. In this dataset, tumours have representation from five distinct anatomic structures (leading edge, infiltrating tumour, cellular tumour, microvascular proliferation, and pseudopalisading cells around necrosis) obtained through laser microdissection of surgically excised glioblastoma tissue. To parallel the sampling location for our single cell analysis, we consider “leading edge” and “infiltrating tumour” to constitute tumour periphery and “cellular tumour”, “microvascular proliferation”, and “pseudopalisading cells” to constitute tumour core. Genes with zero counts across all samples were excluded and normalization was carried out using the “limma”^32^ R package with default parameters.

### Cell clustering and gene-enrichment analysis

**Figure 1:**
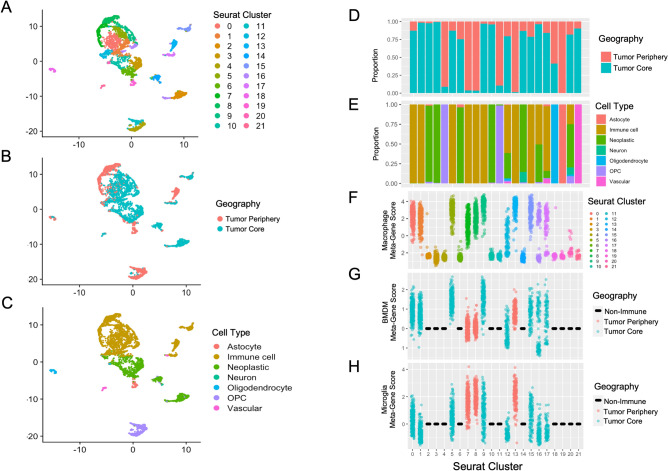
UMAP clustering of single cell data with Seurat. Cells are clustered in 2 dimensions using the UMAP dimensionality reduction technique and annotated by cluster label (**A**), geography (**B**), and cell type (**C**). The relative proportions of cell locations and cell types within each cluster are examined using the stacked barplots in (**D**,**E**), respectively. Meta-gene expression of macrophage, bone-marrow derived macrophage, and microglia-derived macrophage gene signatures by cluster are plotted in (**F**–**H**), respectively. In (**G**,**H**), the meta-gene scores of non-immune clusters are set to 0, core-predominant clusters are coloured blue and periphery-predominant coloured red.

We embedded and clustered our single-cell expression data using *Uniform Manifold Approximation and Projection* (UMAP), a manifold learning approach to dimensionality reduction which has been shown to preserve global data structure better than other similar techniques^33^. This technique is included within the Seurat package; settings were left as default. The resultant cluster map was annotated according to sample location (periphery vs core) and cell type (Fig. 1A–E).

We defined a meta-gene as the difference between the centered, log-transformed mean counts of an input gene list and the centered, log-transformed mean counts of the remaining genes in an array, adopting the approach described by Tirosh et al.^34^. This allowed for interpretation of how a known biological program (rather than an individual gene) varied between cells/tumours. To further explore the UMAP clusters we evaluated the meta-gene expression of a general macrophage gene signature^18^ to compare with known immune cell annotations (Fig. 1F), as well as signatures implicated in bone-marrow-derived macrophages (BMDM) and microglia-derived macrophages^19^ (Fig. 1G,H).

Finally, we used MacSpectrum^10^, a single-cell RNA-sequencing based gene enrichment tool to infer the macrophage activity of our immune population (Supplemental Fig. 1). This technique estimates the Macrophage Polarization Index (MPI) and Activation induced Macrophage Differentiation Index (AMDI) based on input RNA-seq count data; both scores have ranges from -50 to 50. With this score-based approach we were able to map macrophage activity onto a biological spectrum rather than using a categorical representation. A higher MPI value indicates greater pro-inflammatory features and higher AMDI indicates greater maturity. Adopting Li et al.’s approach^10^ in some parts of the analysis, we use zero as a threshold to define “pre-activation” or “M0” cells (AMDI < 0, MPI < 0), “M1-transitional” or “M1 pre-activation” cells (AMDI < 0, MPI > 0), “M2-like” cells (AMDI > 0, MPI < 0), and “M1-like” cells (AMDI > 0, MPI > 0). We note that despite these conventions, the M1/M2 paradigm of macrophage activation remains controversial; M1 is meant to be synonymous with “pro-inflammatory/anti-tumor” and M2 is meant to be synonymous with “anti-inflammatory/pro-tumor”.

### Cell recruitment analysis

**Figure 2:**
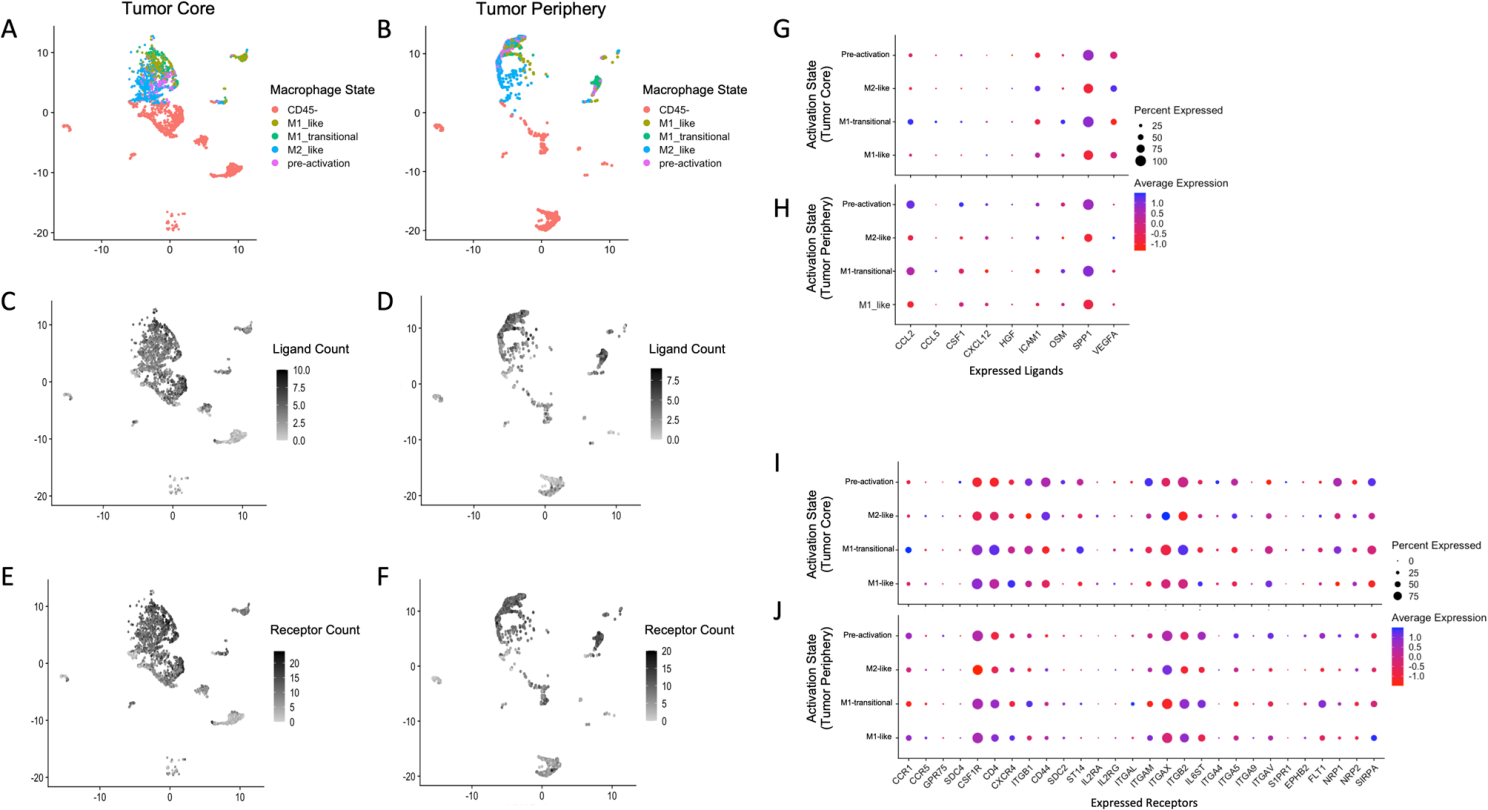
Receptor and ligand expression density by location and macrophage activation state. (**A**,**B**) UMAP plots annotated with macrophage activation state in core and periphery cells, respectively. (**C,D**) The same UMAP plots as above with shade corresponding to the number of filtered ligands expressed by each cell. (**E**,**F**) As above, with shade corresponding to the number of filtered receptors expressed by each cell. (**G**,**H**) Dot plot representation of the proportion of ligands expressed and average expression in tumour core (**G**) and periphery (**H**) by macrophage activation state. I and J: Dot plot representation of receptors expressed in core (**H**) and periphery (**I**) by macrophage activation state.

To attempt to infer cell recruitment patterns in our single cell data, we amalgamated a list of 25 ligands found to be relevant in GBM tumour-associated macrophages (TAMs) based on previous systematic reviews on this topic^35–37^ (Supplementary data). These ligands were filtered to include only those which are expressed by at least 5% of cells^38^ and paired with known receptors using the FANTOM compendium^39^. Resultant receptors were filtered in the same way as the ligands. The ligand and receptor density in both tumour core and periphery was assessed by macrophage activation state (Fig. 2). We also assessed the relative importance of individual ligands and receptors using a hive plot representation (Supplemental Fig. 2). Importance ranking of a receptor in a particular macrophage activation state was computed as being proportional to the sum of edge weights in all loops beginning and ending with the activation state of interest and containing the receptor of interest (Supplemental Fig. 3).

**Figure 3:**
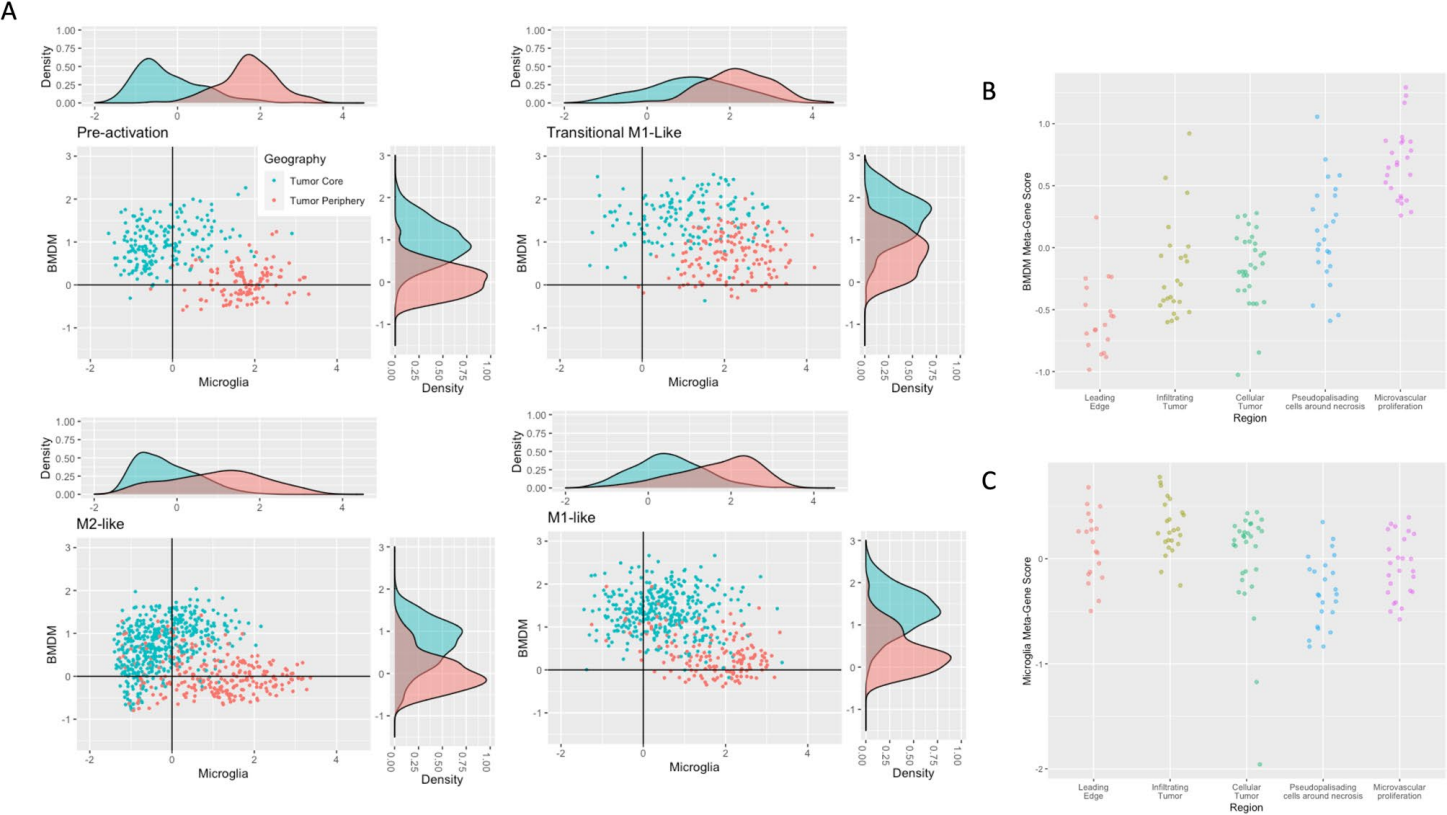
Macrophage recruitment: bone-marrow vs. microglia. (**A**) Scatter plot of microglia-derived meta-gene signature (x-axis) vs. BMDM meta-gene signature (y-axis) for each macrophage activation state. Density plots are used the show the separation in average metagene scores on both axes between core and peripheral cells. (**B**) BMDM scores by anatomical location in bulk validation data. (**C**) Microglia abundance represented using metagene scores by anatomical location in bulk validation.

We further analysed immune cell recruitment in tumor core versus periphery using the meta-gene expression of bone marrow and microglia-derived macrophages^10^ (Fig. 3). The degree of regional separation for each macrophage activation state was assessed with a support vector regression (SVM) model with radial kernel, tuned using tenfold cross validation. The model was trained on a random subset (70%) of the data and performance assessed on the remaining cells. BMDM and microglia-derived macrophage scores for each cell were used as inputs. Bulk data was used as validation using the same approach^22^. Additionally, we compare microglia in the tumor periphery to normal microglia (derived from accession GSE135437 and GSE134705).

Where indicated we used hard thresholding to derive cell identities with the AUCell function from the SCENIC package^40^. Details of the method is described in the reference, briefly the algorithm evaluates the enrichment of a marker gene set in each cell based on area under the curve (AUC) value and a cut-off threshold for these values is used to determine cell identity. Notably, this is the only case where hard-thresholding into microglia/BMDM is used; elsewhere these states are considered to represent the ends of a continuous spectrum.

### Single-cell trajectory analysis and pseudotime ordering

To better understand the landscape of tumour-associated macrophages in tumour core versus periphery we used single-cell trajectory analysis, a machine learning method to embed cells into a linear/branched representation of their gene expression profiles. The aim of this approach is to infer, based on the expression pattern of a gene subset, how cells evolve from one state to another (i.e. from precursor cells to macrophages). Pseudotime, a measure of the overall degree of transcriptional change in this subset of genes, orders cells along a fitted tree structure such that the highest pseudotime values are associated with terminally differentiated states.

We employed Monocle^41,42^ (version 2.14.0) to perform this analysis on core and peripheral cells separately. Following Monocle’s suggested pipeline, genes were considered useable if expressed in at least 10 cells, and cells with outlier total mRNA (outside 2 standard deviations of log10-transformed counts) were omitted. Normalization was done using Monocle’s embedded “Census” method with default settings, which converts scRNA transcript counts (following a negative-binomial distribution) to relative transcript counts. Full details of this method can be found in the paper cited above.

We selected genes to define our cell trajectories using an unsupervised feature selection approach called “dpFeature”, which is included in the Monocle pipeline. Briefly, an array with genes expressed in at least 5% of cells was dimensionality-reduced with t-SNE. This was clustered into relevant cell subpopulations using density peak clustering, a technique which computes each cell’s local density (P) and the nearest distance (D) of a cell to another cell with higher distance. Cells with P and D values above a threshold (default 0.95) are defined as density peaks, each of which defines an individual cluster. Optimal clustering was achieved with 8 clusters in core and 4 in periphery. The most variable genes were extracted by computing likelihood ratios for each gene between a generalized linear model with and without cluster label as a covariate, and the top 1000 genes by q-value were used in the trajectory analyses.

**Figure 4:**
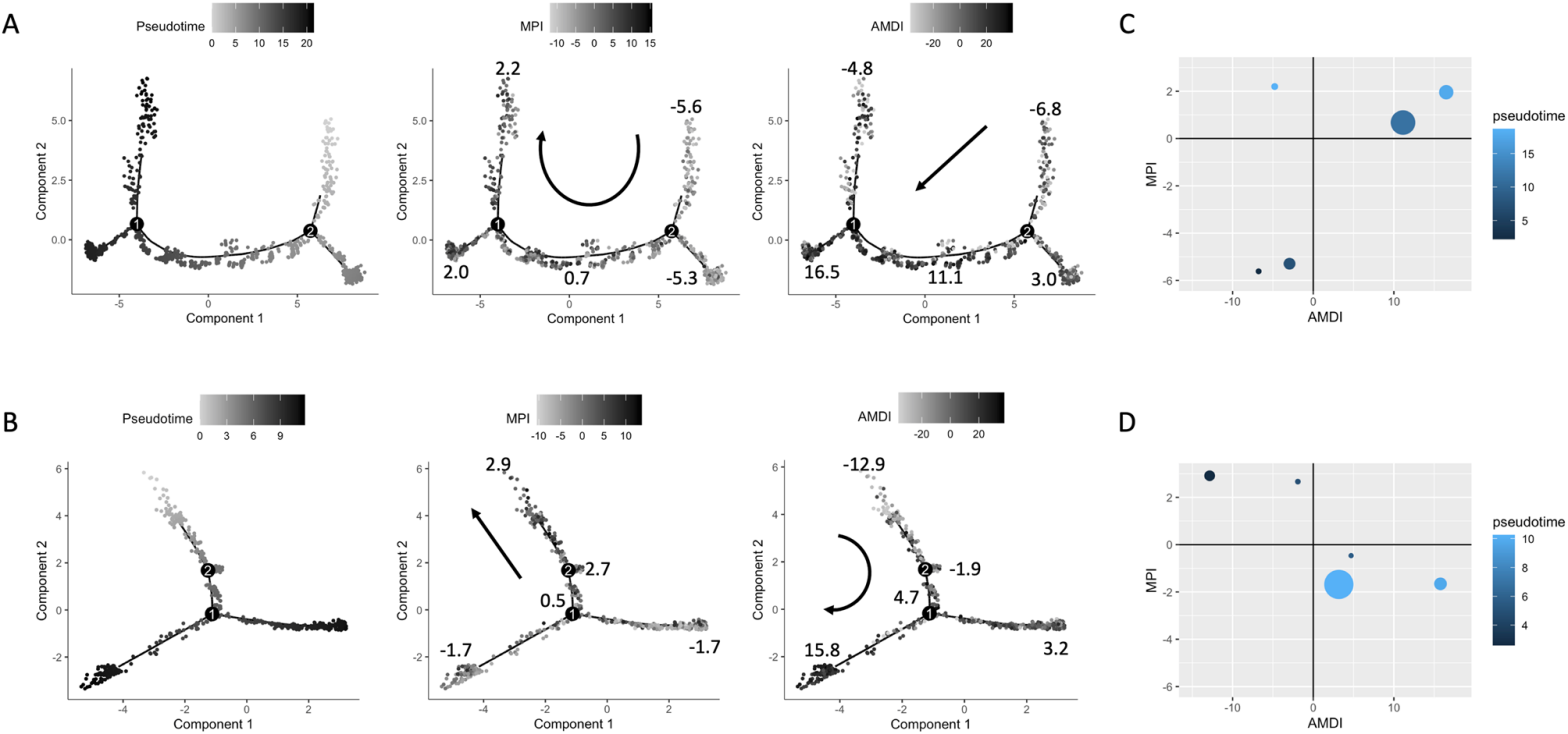
Single cell trajectory analysis and pseudotime ordering. (**A**) Tree structure of tumour core immune cells, coloured by pseudotime, MPI, and AMDI (right to left). Note that there are two branches (labeled 1 and 2) and five states (lines), labelled numerically in the leftmost plot. These states are the result of the unsupervised DDRTree algorithm and represent the evolution pathway of immune cells. Mean scores for each state are labelled in the MPI and AMDI-annotated plots, and an arrow is used to demonstrate the direction of numeric increase. (**B**) The same process is repeated for the peripheral immune cells, whose tree structure also has 2 branches and 5 states. (**C**,**D**) Correlation of mean state-specific AMDI and MPI scores in tumour core (**C**) and periphery (**D**). For each state in the tree structures, mean AMDI and MPI value is plotted, with dot size proportional to the number of cells in that state and colour representing the state’s mean pseudotime value. Numbers correspond to the state labels from the leftmost plots in (**A**,**B**).

Trajectory analysis was carried out using DDRTree^43^ (Discriminative Dimensionality Reduction Tree), a machine learning technique which fits a latent tree structure (in low dimensions) from high dimensional input data. Cells were ordered such that the lowest pseudotime value was set as the terminal cell on the line (“state”) with lowest mean AMDI score. Trajectories were annotated with AMDI and MPI scores to assess their association with pseudotime (Fig. 4).

### Annotation and analysis of branch points

**Figure 5:**
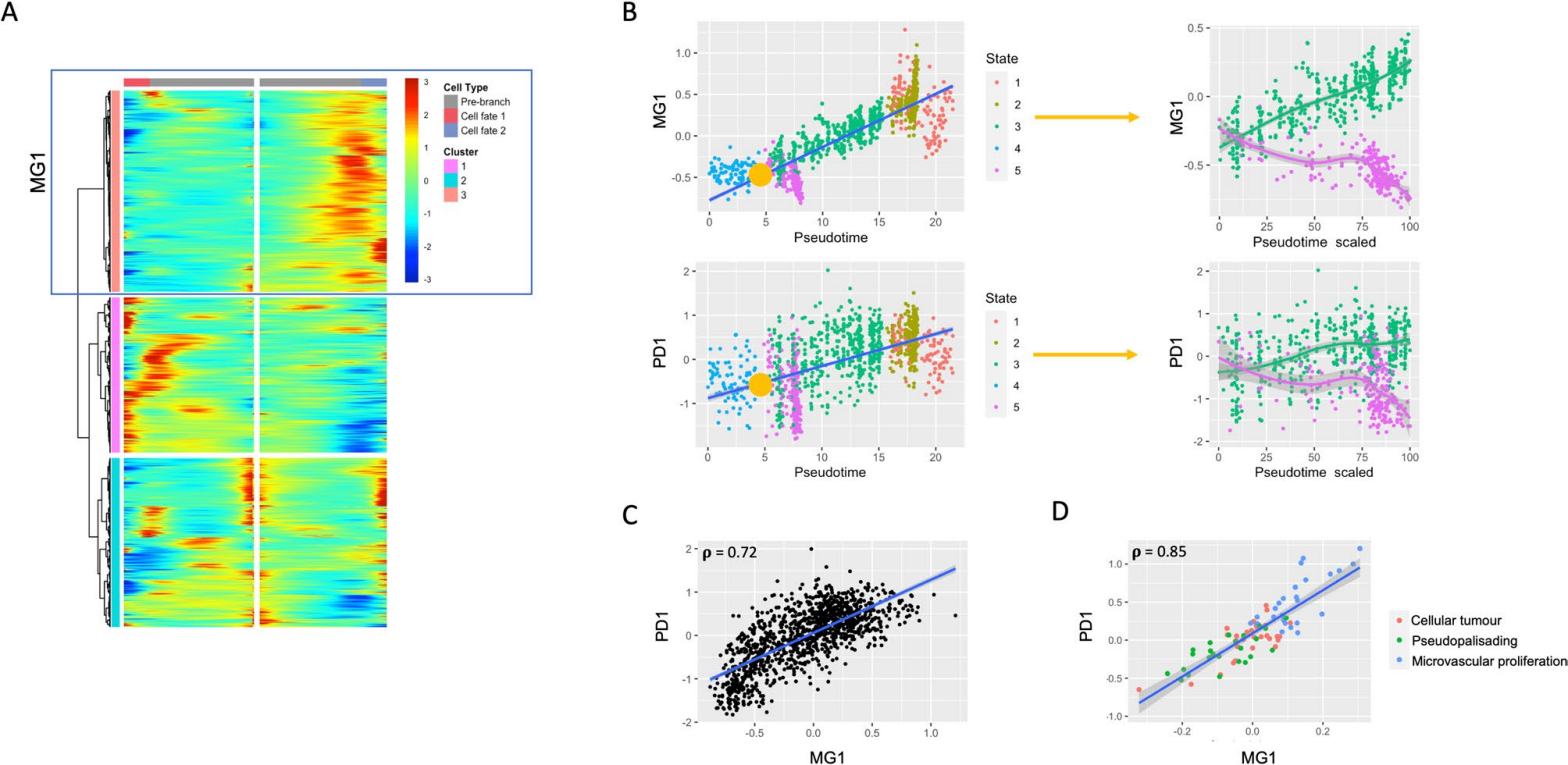
Core tumour cell branch analysis reveals PD-1 associated cell population. (**A**) Heatmap of branch 2-dependent gene expression as a function of pseudotime. Genes constitute rows and pseudotime values as columns. Pseudotime evolves from the middle of the heatmap outwards, with leftward movement from center being associated with increasing pseudotime along output state A (state 5) and rightward movement being associated with increasing pseudotime along output state B (state 3). Hierarchical clustering identifies 3 gene groups with similar pseudotime evolution in a given cell state. Cluster 3 (boxed, labelled MG1) mapped strongly to PD-1 signalling. (**B**) Correlation of MD1 and PD1 meta-genes with pseudotime, annotated by state. Branch 2 is represented by a yellow dot, with input state 4 and output states 3 and 5. The expression of each meta-gene in both output states from branch 2 is shown to the right of these plots, with pseudotime values for each state scaled to a range of 0–100. (**C**) Correlation plot of MG1 and PD1 signalling meta-gene, showing significant positive correlation (Pearson’s correlation 0.72. p < 0.05). (**D**) Validation of MG1 as a marker of PD-1 enriched subpopulation in bulk data from tumor core. Significant positive correlation is noted between meta genes in core samples from bulk data (Pearson’s correlation 0.85. p < 0.05).

**Figure 6:**
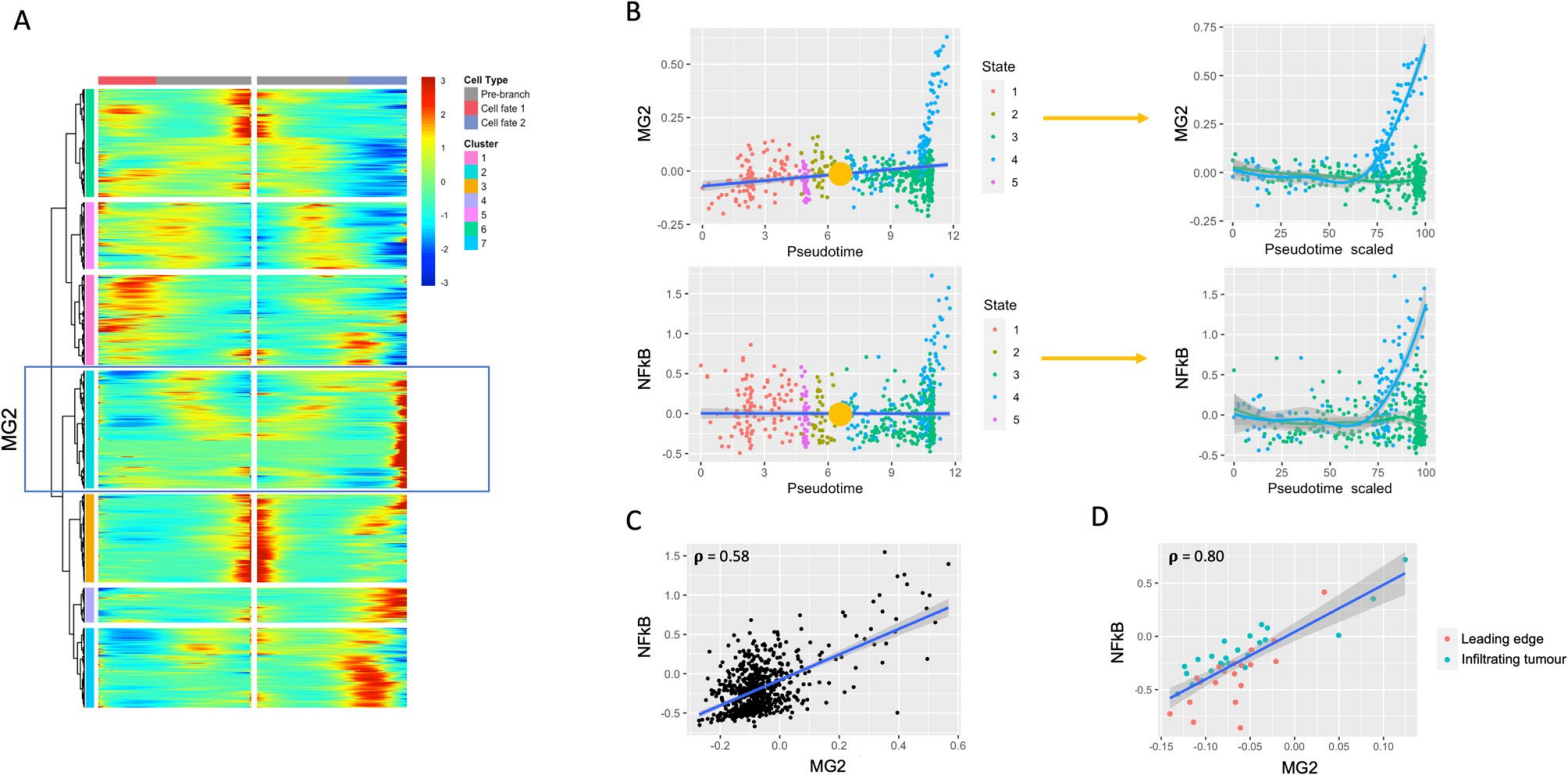
Peripheral tumour cell branch analysis reveals NFkB-associated cell population. (**A**) Heatmap of branch1-dependent gene expression as a function of pseudotime. Heatmap layout as in Fig. 5, with state A corresponding to state 3 and state B corresponding to state 4. Hierarchical clustering identifies 7 gene groups with similar pseudotime evolution in a given cell state. Cluster 2 (boxed, labelled MG2) mapped strongly to NFkB signalling. (**B**) Correlation of MD2 and NFkB meta-genes with pseudotime, annotated by state. Branch 1 is represented by a yellow dot, with input state 2 and output states 3 and 4. The expression of each meta-gene in both output states is shown to the right, with pseudotime values for each state scaled to a range of 0–100. (**C**) Correlation plot of MG2 and NFkB signaling meta-gene, showing significant positive correlation (Pearson’s correlation 0.58. p < 0.05). (**D**) Validation of MG2 as a marker of NFkB enriched subpopulation in bulk data. Significant positive correlation is noted between meta genes in tumour periphery samples from bulk data (Pearson’s correlation 0.8. p < 0.05).

Since branch points in the trajectory analysis (nodes containing one input state and two output states) are likely to represent important “decision points” during macrophage evolution, we used Monocle’s BEAM function to compute likelihood ratios between the expression of each gene in both output states. Genes with q-values < 0.05 were considered “branch-dependent”. Hierarchical clustering of each branch-dependent gene (for each branch) was used to subset the genes into subgroups with similar pseudotime evolution. Gene clusters with strongly divergent expression (whose meta-gene expression differed strongly between terminal cells in both output states) were probed for potentially relevant mechanisms using *Enrichr*^44,45^. A meta-gene constructed from the cluster of genes from which important signaling were found was investigated as a function of pseudotime and correlated to the meta-gene constructed from a signature of the mechanism itself. This latter meta-gene correlation was verified using bulk data (Figs. 5 and 6).

### Computational platform

All analysis was carried out using R^46^ (version 3.6.3).

## Results

### Data demographics

We used single cell RNA-seq data obtained from Gene Expression Omnibus^26^ accession GSE84465^7^, which consists of 3589 cells obtained from 4 glioblastomas, each with representation from tumour core (2343 cells) and periphery (1246 cells) (Table 1). Most cells were labeled as immune (51%, 1847 cells) or neoplastic (30.4%, 1091 cells), with the remained divided amongst oligodendrocyte-precursors (OPCs), astrocytes, oligodendrocytes, vascular cells, and neurons in decreasing proportions. Notably, after subclassification with Macspectrum, the immune population consisted of 1160 cells from tumour core and 646 cells from tumour periphery (Table 2). Applying MacSpectrum to the tumor core immune subpopulation yielded 462 (40%) are M2-like, 358 (31%) are M1-like, 188 (16%) are M0, and 152 (13%) are M1 pre-activation cells. In the periphery, applying the same approach, 235 cells (36%) are M2-like, 156 (24%) are M1-like, 139 (22%) are M1 pre-activation, and 116 (18%) are M0. No significant differences were noted between the proportion of M2-like or M0 cells between core and periphery, though tumour core contains significantly more M1-like cells (31% vs. 24%, Chi-squared p = 0.003) and fewer M1 pre-activation cells (13% vs 22%, Chi-squared p < 0.0001) than periphery. No difference was noted between the total number of pro-inflammatory cells (M1-like + M1 pre-activation) between groups, but the peripheral tissue contained a greater number of immature immune cells (M0 + M1 pre-activation; 39% vs 29% Chi-squared p < 0.0001).

**Table 1:** Single cell data cell characterization.

Cell type	All N (%)	Core N (%)	Periphery N (%)	P*
All cells	3589 (100)	2343 (100)	1246 (100)	N/A
Immune	1847 (51.5)	1182 (50.4)	665 (53.4)	0.103
Neoplastic	1091 (30.4)	1029 (43.9)	62 (5.0)	< 2.2 × 10^–16^
OPC	406 (11.3)	50 (2.1)	356 (28.6)	< 2.2 × 10^–16^
Astrocytes	88 (2.5)	0 (0)	88 (7.1)	< 2.2 × 10^–16^
Oligodendrocytes	85 (2.4)	34 (1.5)	51 (4.1)	1.30 × 10^–6^
Vascular	51 (1.4)	47 (2.0)	4 (0.3)	9.15 × 10^–5^
Neurons	21 (0.6)	1 (0.0)	20 (1.6)	1.99 × 10^–8^

*Chi-squared p value comparing core and periphery.

**Table 2:** Immune activation states of single cell data based on Macspectrum.

Activation	All cells N (%)	Core N (%)	Periphery N (%)	P*
All	1806 (100)	1160 (100)	646 (100)	N/A
M1-like	514 (28)	358 (31)	156 (24)	0.003
M2-like	715 (40)	462 (40)	253 (36)	0.82
M1 pre-activation	291 (16)	152 (13)	139 (22)	< 0.0001
M0	304 (17)	188 (16)	116 (18)	0.38

*Chi-squared p value comparing core and periphery.

Bulk validation was carried out with the publicly available Ivy Glioblastoma Atlas Project^22^, comprised of a total of 122 RNA samples from 10 different tumours with sampling from five disctinct regions (leading edge, infiltrating tumour, cellular tumour, microvascular proliferation, and pseudopalisading cells around necrosis). This cohort contains entirely primary GBMs that underwent gross total surgical resection. Median age is 57.5 years (range 18–67), 6/10 are males, and median survival is 871 days (range 105–1293). Most are IDH wild type (9/10), with a single tumour having IDH1 mutation. Additionally, half of tumours (5/10) have positive MGMT methylation status. Tumours are also labelled according to molecular subgroups: 2 classical, 3 proneural, 1 neural, 2 classical/neural, and 2 classical/mesenchymal.

### Single cell gene expression programs define uniform immune subgroups in human GBM

Single cell clustering of all 3589 cells using Uniform Manifold Approximation and Projection (UMAP, see “Methods”) identified 22 distinct cell clusters (Fig. 1A). There was good separation of tumour location, with limited mixing of cell locations within clusters (Fig. 1B,D). Additionally, there is strong geometric separation of cell types suggesting the validity of the clusters obtained (Fig. 1C,E). We noted that within the immune cell clusters there is consistent upregulation of our macrophage meta-gene score (Fig. 1F) and that the average bone marrow-derived macrophage meta-gene score is higher in tumour core clusters whereas the average microglia-derived macrophage meta-gene score tends to be higher in periphery-dominated clusters (Fig. 1G,H). This is in keeping with the findings of Müller et al.^19^ from their single cell analysis 13 untreated primary gliomas. Macrophage activation states mapped reasonably well onto cell clusters (Supplemental Fig. 1) and identified, for example, several subgroups enriched in either low or high inflammatory activation (M0 and M2 or M1 pre-activation and M1, respectively). We also found that the expression profile of peritumoral microglia is distinct from that of homeostatic microglia, which cluster together across different studies (Supplementary Fig. 9A,B). Furthermore, of the two modules discovered via WGCNA, one of them (the “blue” module) is distinctly expressed by only peritumoral microglia (Supplementary Fig. 9C). Using EnrichR, we found that this module enriches in linoleic acid metabolism and VEGF-related pathways using the REACTOME 2016 database (Supplementary Fig. 9D).

### Tumor associated macrophages follow distinct recruitment patterns in human GBM

Based on prior observations and our own findings, we hypothesized that immune cells follow distinct recruitment patterns in GBM. We firstly sought to understand the number of salient tumour-associated macrophage ligands proposed to be involved in the recruitment of TAMs (derived from published systematic reviews^35–37^) and their paired receptors using the FANTOM dataset^47^. In our exploratory analysis we found that several receptor-ligand interactions proposed to be implicated in TAM recruitment appear to be expressed amongst all TAM activation states, and note a particularly important role of M1-transitional cells in the periphery, and of pre-activation cells and M1-transitional cells in the core with considerable overlap (Supplemental Fig. 2). Notably, the top ranked ligands in both core and periphery are SPP1, ICAM1, and VEGF-A. The top 3 ranked receptors in core are ITGB1, ITGB2, and CD44, while the top ranked in periphery are ITGB2, ITGAX, and CSF1R. We subsequently took an approach that would demonstrate the parallel signalling processes within each cell. We firstly examined the expression density of receptors and ligands from both core and peripheral tumour cells (Fig. 2). As expected, we found multiple ligands (mean 3.2, SD 2.0) and receptors (mean 9.0, SD 4.7) expressed per cell, evidencing parallel biological processes underlying TAM recruitment. Notably, the mean number of ligands expressed by immature macrophage states (M0 and M1 pre-activation cells) was significantly higher than mature macrophage states (M1-like and M2-like) in both core and periphery (4.5 vs 3.8, Mann–Whitney p = 0.001 and 4.7 vs. 3.8, M–W p < 0.0001, respectively). The same was noted for the mean number of receptors expressed (13.7 vs. 11.4, M–W p = 0.0004 and 11.0 vs. 9.4, M–W p < 0.0001, respectively). Additionally, the average number of both receptors and ligands expressed in pro-inflammatory states (M1 pre-activation and M1-like) was significantly higher than the average in anti-inflammatory states (M0 and M2-like) for both core and periphery (MW p < 0.0001). We then focused on the regional differences in the pool of pre-activation cells which would represent TAM’s at the more initial stages of recruitment. We inferred dominant receptor activation in these cells by ranking them based on relative weight of receptors (Supplementary Fig. 3). We found receptor components integrin subunit beta 1 and alpha V were top ranked in both core and periphery. Receptors CD44 and NPR1 had considerably greater inferred activity in tumor core (ranked #3 and #4, respectively, in core and #6 and #15, respectively, in periphery) whereas CSF1R and CCR1 were considerably more abundant in receptor periphery (ranked #5 and #3 ,respectively, in periphery and #17 and #16, respectively, in core).

We next investigated the ontogeny of TAMs in different anatomical locations. We compared the abundance of TAMs with bone marrow (BMDM) versus microglia origin by scoring each cell based on their expression of marker genes as described in the Methods. These relative BMDM and microglia-specific expression scores demonstrate clear differences between the immune cell populations of core and periphery. Specifically, BMDMs were consistently more prevalent in the core whereas microglia-derived TAMs were more prevalent in the periphery (Fig. 3). Notably, an SVM classifier yielded high area under the receiver operating curves (AUCs), ranging from 0.83 in transitional M1-like cells to 0.99 in pre-activation (M0). There was a significant difference between the classifier performance in M0 cells and all other individual curves (DeLong’s p < 0.05), and the three remaining curves were statistically similar to one another (Supplemental Fig. 4A). Validating these results in bulk tumour samples derived from different anatomical locations, we achieve an AUC of 0.98 with the same approach (Supplemental Fig. 4B).

### Trajectory analysis shows distinct regional inflammatory states for tumor associated macrophages

We next sought to investigate the dynamics of macrophage maturation in tumor core and periphery. Rather than comparing fixed cell states, we carried out single cell trajectory analysis to explore intermediate cell states and their molecular drivers and identify important transcriptomic branch points relevant to this process. Cells were ordered on a fitted tree model by computing a pseudotime value based on the expression profile of highly variable genes. This pseudotime value is designed to represent the biological state of each cell along a pathway towards maturation. Notably, this approach assumes that although the tumour sample was taken at a specific time, it captures cells in different biological states which, in a longitudinal analysis, would shift from one state to another. Both core and peripheral immune cells yielded tree structures consisting of 5 states (lines) and 2 branches (Fig. 4A,B). Expectedly, the immune cell maturity score (AMDI) was positively associated with pseudotime in both core (Pearson correlation 0.29, p < 0.0001) and periphery (Pearson correlation 0.41, p < 0.0001). However, the macrophage polarization (MPI) was positively correlated with pseudotime in the core sample (Pearson correlation 0.46, p < 0.0001) but inversely associated in the periphery sample (Pearson correlation − 0.34, p < 0.0001) (Fig. 4C,D; Supplemental Fig. 5). This suggests that cells within the tumour core tend towards increasingly pro-inflammatory features as they mature, whereas cells within the tumour periphery tend towards increasingly anti-inflammatory features.

### Branch analysis reveals PD-1-associated subpopulation in tumour core and NFkB-associated subpopulation in tumour periphery

For each branch point in the trajectory analyses, we identified branch-dependent genes whose expression as a function of pseudotime was significantly different between output states (likelihood ratio q-value < 0.05, see methods for further description). Genes were clustered using hierarchical clustering, and groups with divergent mean expression between the two output states were investigated for enrichment in potentially important mechanisms.

One cluster of branch 2-dependent genes from the tumour core analysis enriches strongly in PD-1 signaling (Fig. 5A). A meta-gene constructed from this gene list (meta-gene 1; MG1, comprised of 756 genes) strongly differs in expression as a function of pseudotime between the two output states of branch 2: increasing with pseudotime in state 3 and decreasing with pseudotime in state 5. A similar pattern is observed using a meta-gene constructed from known PD-1 signaling transcripts (Fig. 5B). Additionally, a significant (p < 0.05) positive correlation is observed between these two meta-genes (MG1 and PD1) both in single-cell data (Fig. 5C; Pearson correlation 0.72, p < 0.05) and bulk validation core data (Fig. 5D; Pearson correlation 0.85, p < 0.05). Importantly, MG1 may define a relevant subpopulation of cells within tumour core that is strongly associated with PD-1 signalling. It appears as though there may be an important branch point early in immune cell maturation where this population is negatively selected, as evidenced in state 5. Annotation of all other core branch-specific gene clusters can be found in Supplemental Fig. 6.

In our analysis of tumour periphery, we find a branch 1-dependent gene cluster which maps strongly to NFkB signaling (Fig. 6A). This meta-gene (MG2, comprised of 340 genes) does not vary strongly with pseudotime, but is very strongly expressed in the terminal cells of state 4 (one of two mature states). A similar pattern is found using a metagene constructed from known NFkB signalling transcripts (Fig. 6B). A significantly positive correlation is found between MG2 and NFkB signaling in both single cell data (Fig. 6C; Pearson correlation 0.58, p < 0.05) and bulk validation data from peripheral tumour samples (Fig. 6D; Pearson correlation 0.80). This suggests a strongly NFkB-dependent subpopulation of cells in the periphery, whose positive selection appears to occur late during the immune maturation process. Annotation of all other periphery branch-specific gene clusters can be found in Supplemental Fig. 7.

## Discussion

### Overview of results

In this study we investigated several important aspects of tumor associated macrophages in GBM, namely their origin, recruitment, activation states and plausible driver mechanisms. Our findings demonstrate biological redundancy in TAM recruitment mechanisms. Furthermore, we found that TAMs in the tumour core mostly originate from the bone marrow derived pool whereas those in the tumour periphery are largely derived from microglial cells, supporting prior research^19^. Using trajectory analysis with gene enrichment analysis we found differing profiles of macrophage activation by tumour region (core vs. periphery). Interpreting our findings using the conventional model for macrophage activation we find that cells in tumor core evolve from a “pre-activation” state towards pro-inflammatory state. However, this seemingly oncotoxic process is paralleled by increasing activity of PD-1-signaling, a known mechanisms of immune silencing in tumors and a target for immune checkpoint blockade^48^. In tumor periphery, by contrast, we found that cells transition from a pre-activation state towards pro-oncogenic activation (M2 or “alternative” activation state). We find this trajectory of cells also contains an important subpopulation with strong NFkB signaling, highlighting a potential mechanism through which to reprogram periphery-resident TAMs^49^. To our knowledge, this study is the first to analyze geographical differences in macrophage recruitment and activation through sequential activation states. These findings may have implications on immune-based treatments of GBM, and in particular we identify a potential mechanism of resistance to immune checkpoint blockade in tumor periphery.

### TAM ontogeny in GBM

Understanding the origin and recruitment of TAMs in GBM is fundamental to understanding the immune landscape of these tumors and to developing immune modulation strategies. The two currently proposed sources of TAMs are bone marrow derived monocytes (BMDM), which reach the tumor through the blood stream, and CNS-resident microglia cells activated locally by the tumor microenvironment. The identification and tracking of these cell pools has been problematic due to the lack of consistent markers, and a number of studies using different models have disagreed upon the relative proportions of each in GBM^50–53^. Our approach uses gene expression programs, rather than single markers, to represent cell identity as a numeric score in order to reflect the biological continuum of in vivo cells. Another hurdle for translation is the heterogeneity of GBM cell composition which contains distinct histological units such a cellular, necrotic, hypervascular, invading and leading edge^22^. Samples from different regions will therefore capture distinct cellular features and biological processes. In keeping with this concept, we find TAMs in our combined single cell and bulk analysis that TAMs in the tumor core predominantly originate from BMDMs while in tumor periphery they are derived most from microglia.

### TAM recruitment in GBM

The driving mechanisms of TAM recruitment have largely been studied in cell cultures or animal models with implanted glioma cell lines. Most studies test single mediators/receptors such as MCP-1^54^, MCP-3^55^, CXCL12^56^, CX3L1^57^, CSF1^58^, LOX^59^, HGF^60^, Periostin^61^, osteopontin^62^ and others^3,4,35,37,63^ which have shown promise. Importantly, we find that despite their limited numbers, the neoplastic cells in the tumor periphery are associated with osteopontin secretion which is known to facilitate a pro-tumor microenvironment. These results are key to understanding the building blocks of cellular interactions in GBM. In this study we evaluate a host of ligands suspected to be implicated in GBM TAM recruitment as well as their inferred receptors, an approach which has been implemented to infer changes in receptor-ligand interactions between endothelia cells and astrocytes in the aging brain^64^ and decipher the cellular interactome of the lung^38^. Our results suggest parallel receptor-ligand interactions involved in TAM recruitment which may serve as a salvage mechanism through which GBM is able to bypass immune modulation^65^. Nevertheless, we also note that integrin activation appeared as a universal mechanism detected in pre-activation cells of tumor core and periphery, while CD44 and NRP1/2 activity was higher in tumor core and chemokine receptors CCR1 and CSF1R were higher ranked in the tumor periphery. These findings are the first to demonstrate the biological redundancy of receptor-ligand interactions in human GBM tissue and thus promote the need for a combined treatment approach.

### Dynamic identity of TAM in GBM

The classic model of macrophage activation states is derived from in-vitro stimulation experiments^66,67^ wherein IL4 is noted to shift peritoneal macrophages to a state with enhanced endocytotic clearance, reduced antigen presentation and lower inflammatory cytokine production. This state was coined “M2”, or alternative activation^67^ in contrast to the “M1” or classic activation state characterized by pro-inflammatory functions^68^. This model was adopted in the study of tumor immune microenvironment, wherein M1 macrophages are considered “anti-tumor” and M2 as “pro-tumor”, promoting tissue remodelling, angiogenesis^3,61,63^ and malignant growth^62^. However, it has become increasingly recognized that the conventional model of M1 vs M2 macrophages does not translate well to *in-vivo* pathological states^9^. For example, canonical M1 and M2 markers are co-expressed by individual immune cells in traumatic brain injury^69^ as well as gliomas^19^. Furthermore, there is growing evidence that macrophage activation is dynamic and may be context and disease dependent, prompting expansion beyond a binary model^11^. In our analysis, we implemented a continuous score-based approach by Li et al. to characterize each TAM in terms of maturity and inflammatory features^10^. We also modeled the dynamic states of tumor associated macrophages with pseudotime^70^, which infers the position of each cell along a biological continuum based on cross-sectional expression data. Notably, TAM maturity correlated positively with pseudotime in both tumor core and periphery, yet the degree of inflammation was negatively correlated in tumor periphery, suggesting a potential mechanism of immune silencing. This trend may be explained in part by a cell population in tumour periphery with high pseudotime and strong NFkB signalling, which has been previously shown to associate with pro-tumor macrophage activity^49^. In tumor core we find an opposite trend towards a “pro-inflammatory” state. This process correlates with PD-1 signalling in most immune cell subpopulations of the tumour core. These opposing trends may follow from regional differences in selection pressures, and lends to the notion that that pro-inflammatory phenotypes may not be oncotoxic in the core. While there is need to further explore the role of TAMs in GBM, it is becoming increasingly clear that the immune landscape differs strongly between infiltrating and central regions.

### The role of PD-1 in tumor associated macrophages

Programed cell death protein 1 (PD-1) is thought to be a key mediator of tumor-induced immunosuppression, though to date its role is largely understood to be via the regulation of T cells. However, there is emerging evidence that PD-1 is also expressed by tumor associated macrophages^16^, making it a plausible target in GBM TAMs. According to Filley et al.^15^, PD-1 blockade has shown promise in mouse models of GBM. However, their trial (CHECKMATE 143) comparing nivolumab (an FDA-approved PD-1 inhibitor) to bevacizumab (a VEGF-A inhibitor) in recurrent glioblastoma demonstrated only an 8% response rate to nivolumab compared to a 23% response to bevacizumab (yet the duration of response was higher in the nivolumab arm, median 11.1 vs 5.3 months). Several potential reasons for failure are discussed, including an intact blood brain barrier, intra-tumoral T-cell anergy, and global immune dysfunction in patients with high grade glioma. However, the encouraging result in a small subset of patients suggests immune checkpoint inhibition may indeed play a significant role in GBM treatment when combined with adjuvant strategies, and several other trials are now underway. Our study identifies a gene program which is closely associated with PD-1 signalling in macrophages of tumor core. Notably, a small subpopulation of core TAMs appear to inhibit the expression of this program, and may thus by partly responsible for resistance to PD-1 blockade in the majority of patients.

### The role of NFkB in GBM

Nuclear factor kappa-B (NFkB) is a ubiquitous transcription factor involved in the regulation of several immune chemokines. Previous work on mouse models^49,71^ demonstrate considerable promise for the inhibition of this pathway in GBM treatment. Specifically, it has been found that NFkB signalling in involved in the invasive properties of GBM via metalloproteinases, which can be deprecated with NFkB inhibition^71^, and that NFkB knockout mice exhibit decreased tumour growth and increased pro-inflammatory cytokines^49^. Our study identifies a subpopulation of cells in tumour periphery defined by a genetic program that correlates strongly with NFkB signaling in both single cell and bulk peripheral GBM tissue. We note that this program is highly expressed only within a subpopulation of cells in the tumour periphery, suggesting the possibility that this subset of cells plays a critical role in invasion. Further, as NFkB is suggested to drive TAMs towards a more anti-inflammatory environment, it is plausible that this subpopulation plays a role in the observed peripheral cells’ evolution towards an anti-inflammatory environment. Furthermore the enrichment of other transcription factors from the same cluster (BATF, PLXNC1, MTF1) as NFkB have been shown to upregulate in other cancers^72–74^ suggesting NFkB to exert and a pro-tumor effect.

### Study limitations

This study is designed to explore the complex immune microenvironment of GBM and yield novel hypotheses that warrant further in-vitro and in-vivo testing. Consequently, there are a number of limitations that must be considered. Firstly, the use of publicly available data limits our access to additional demographic annotations which may further educate out results. A lack of additional openly-available scRNA data with regional tumor annotation may limit generalizability. Additionally, Macspectrum gene-enrichment programs upon which we classify macrophage activation states were originally derived from BMDM only. This is also true of the other gene signatures used in the paper given a lack of robust gene signatures derived directly from GBM tissue. Nevertheless, we would argue that the relevant transcriptional profile, regardless of cell species, remains a central determinant of biological activity.

### Future directions

This work adds to a growing body of literature suggesting fundamental regional differences in the immune microenvironment of glioblastoma. It is becoming increasingly clear that a treatment that is sufficient in the core may not be sufficient in the periphery and vice versa. While regional differences in blood brain barrier permeability and the sparing of peritumoral brain during resection and radiotherapy remain key reasons for this differential treatment response, differences in immune microenvironment must also be considered. Therefore, it will be important that future studies of glioblastoma microenvironment continue to probe for regional differences in this tumour. Importantly, since current treatments (surgery, radiation) predominantly target tumor core, it will be particularly relevant to develop novel therapeutics targeting the residual invasive periphery to prevent recurrence and spread. Additionally, we suggest the importance of critical immune subpopulations which may influence treatment response. Moving forward, we hope to further explore the role of macrophage evolution in glioblastoma, particular under the influence of PD-1 and NFkB signalling, with large-scale collection of primary and recurrent human glioblastomas. We also envision pairing these data with serum transcriptomic data to identify features of a patients underlying immune status which may correlate to treatment response.

## Conclusions

This study uses recently developed machine learning techniques to further explore the highly complex immune microenvironment of glioblastoma using both single cell and bulk RNA sequencing. We find distinct activation and maturation processes between tumour core and periphery. Our results show bone-marrow derived macrophages evolve towards a pro-inflammatory state defining the core and microglia-derived macrophages evolving towards an anti-inflammatory state defining the periphery. We also identify potentially relevant cell subpopulations in tumor core with variable association to PD-1 signaling one of which may represent a resistant population. We also find NFkB signaling associated with TAM maturation in a cell population of tumor periphery. This holds some promise in the future of GBM treatment. This work advocates for the need for a multi-target molecular approach to GBM therapy and suggests that future research continue to take these regional immune subpopulations into consideration.

## Supplementary information

Supplementary Information 1.

Supplementary Information 2.
