# Supplementary material for: Distinct regional ontogeny and activation of tumor associated macrophages in human glioblastoma
Source: Sci Rep. 2020 Nov 11;10:19542. doi: 10.1038/s41598-020-76657-3 (PMC7658345; doi:10.1038/s41598-020-76657-3)
Supplement: Supplementary file 1 — Supplementary Information 1. [file 41598_2020_76657_MOESM1_ESM.pdf]

SUPPLEMENTAL FILES: Distinct regional ontogeny and activation of tumor associated macrophages in human glioblastoma

Alexander Landry[1], Michael Balas[1], Saira Alli[1], Julian Spears[1], Zsolt Zador[1]

1) Division of Neurosurgery, Department of Surgery, St. Michael's Hospital, Toronto, ON

Corresponding authors:

Alexander Landry

Email: alex.landry@mail.utoronto.ca

Zsolt Zador

Email: zadzso@gmail.com

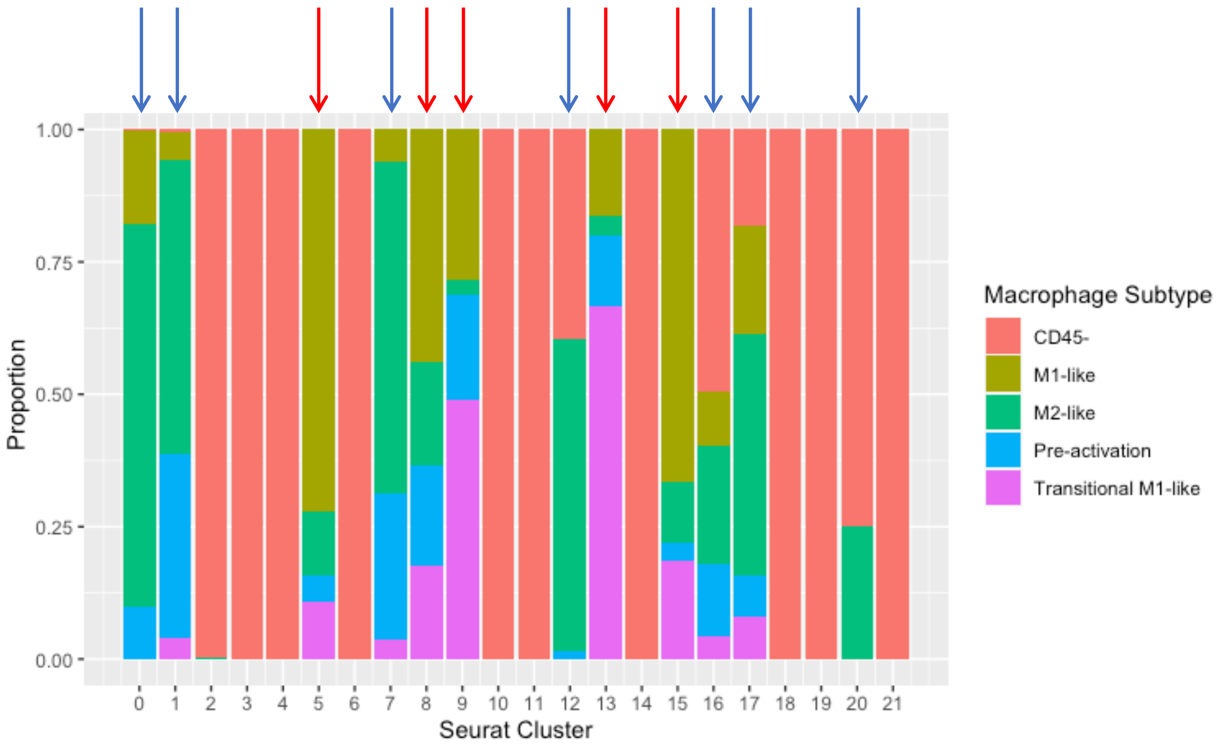

Supplemental Figure 1: Proportion of TAM activation states across UMAP clusters from Figure 1. Clusters containing an immune cell fraction are labeled as pro-inflammatory (red arrows) or anti-inflammatory (blue arrows) based on their relative proportions of pro- (transitional M1-like and M1-like) and anti-inflammatory (pre-activation and M2-like) species.

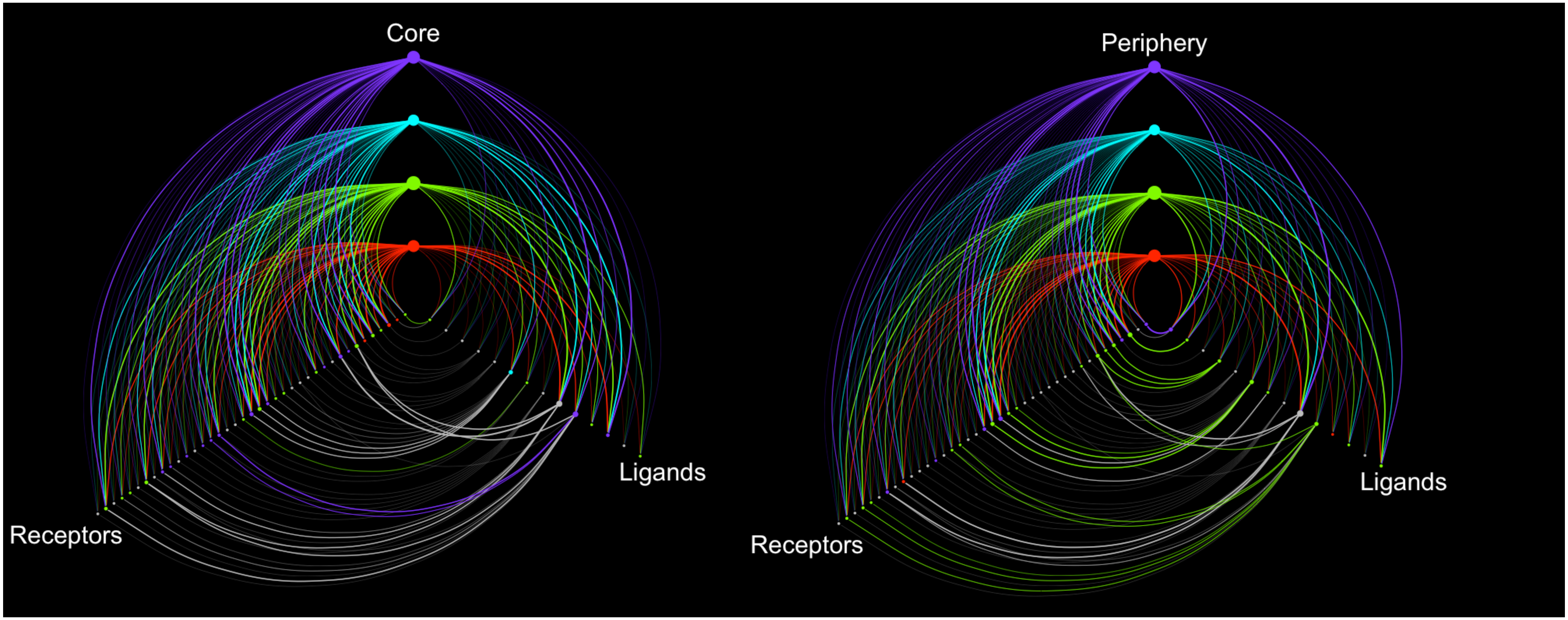

Supplemental Figure 2: Hive plots of TAM-associated receptors and ligands in tumor core and periphery immune cells. Macrophage activation state is plotted on the vertical axis (purple = pre-activation, turquoise = M2-like, green = M1-transitional, red = M1-like). Edge weights between cells and ligands, and between receptors and cells, are proportional to the number of cells expressing the ligand/receptor. Edge weights between ligands and receptors are proportional to the number of receptor/ligand pairs expressed. Node sizes are proportional to the sum of incoming and outgoing edge weights. Ligand and receptor node color reflect the strongest cell association (highest edge weight linking to cells), with ties being colored gray. Edges connecting directly to a cell are colored according to cell type. Edges between ligands and receptors are colored the same as outgoing and incoming nodes only if the nodes are the same color. Otherwise they are colored grey.

A

B

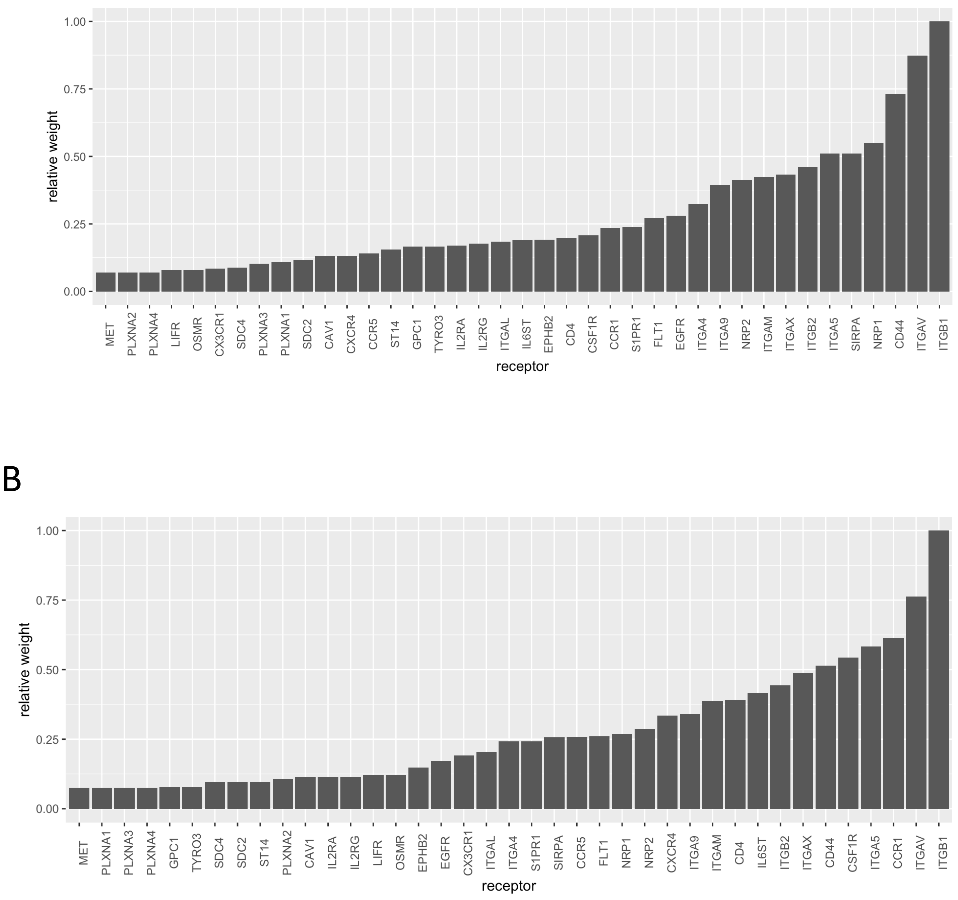

Supplemental Figure 3: Relative weights of TAM recruitment related receptors in pre-activation (M0) cells in core (A) and periphery (B).
Weights of a given receptor are obtained by summing over all loops in the Hive plot (Supplemental Figure 2) which contain pre-activation cells and the receptor.

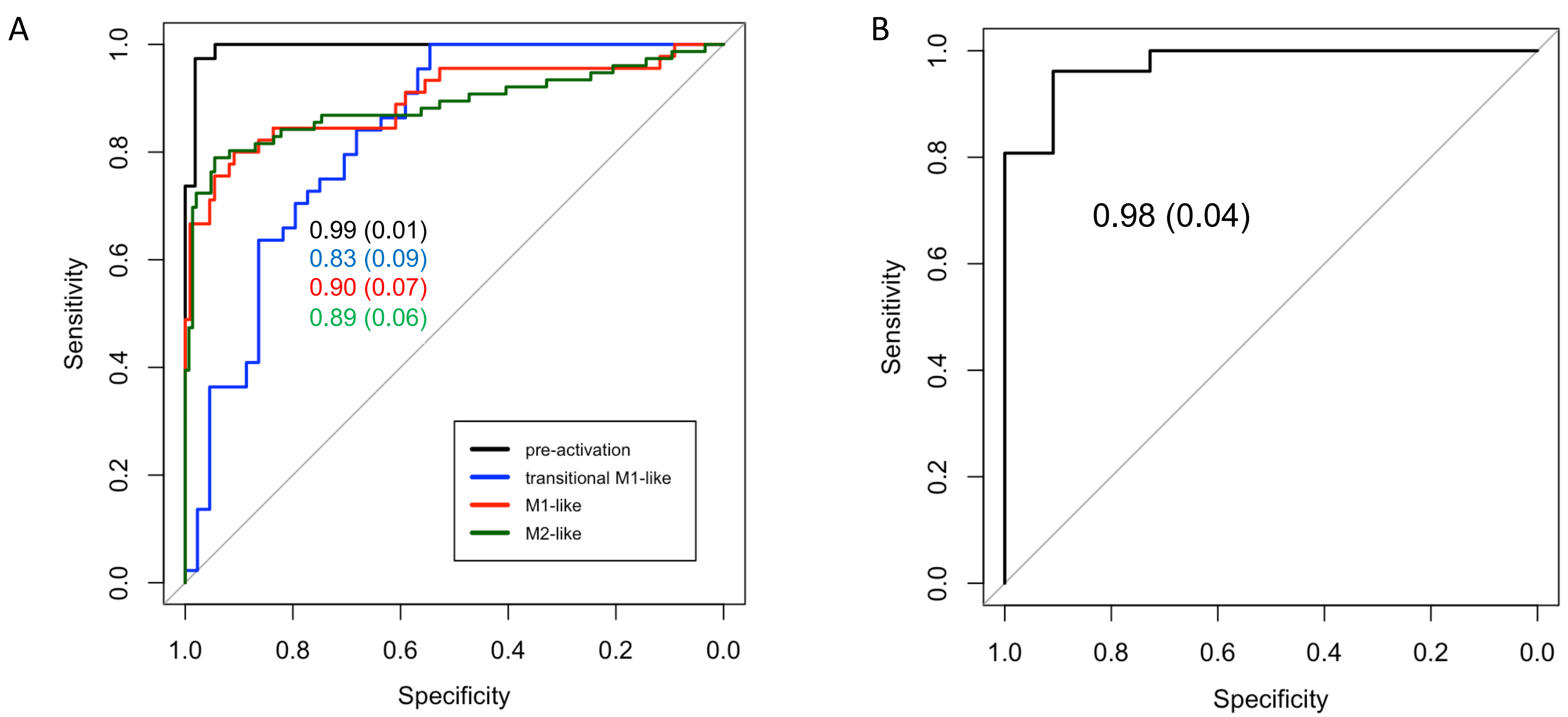

Supplemental Figure 4: SVM classifier performance. A: Receiver operating characteristic curves for each macrophage activation state in single cell data, with microglia-derived macrophage and BMDM scores used as inputs and tumour geography as output.
Areas under the curve and 95% confidence intervals are plotted on the graphs.
Notably, there is a significant difference in performance between pre-activation cells and all other curves (De-Long's $p<0.05$), but no differences between any remaining curves. B: Classifier performance applied to Ivy bulk validation data.

A

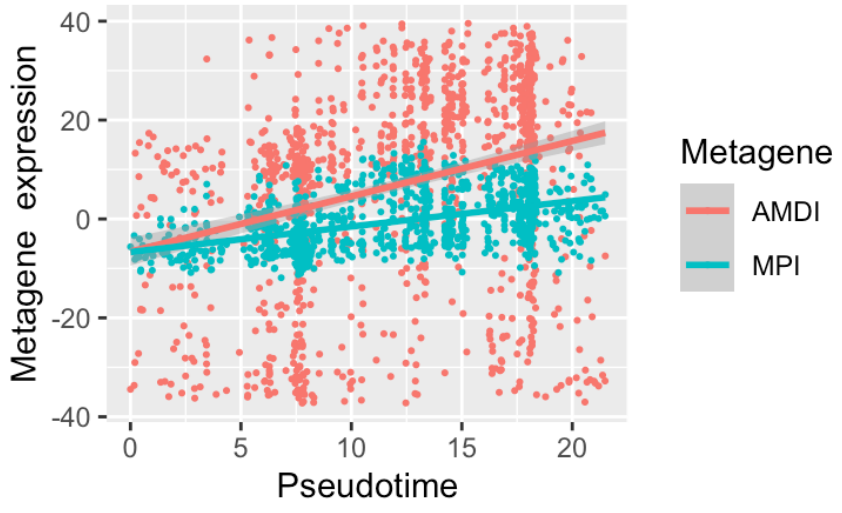

B

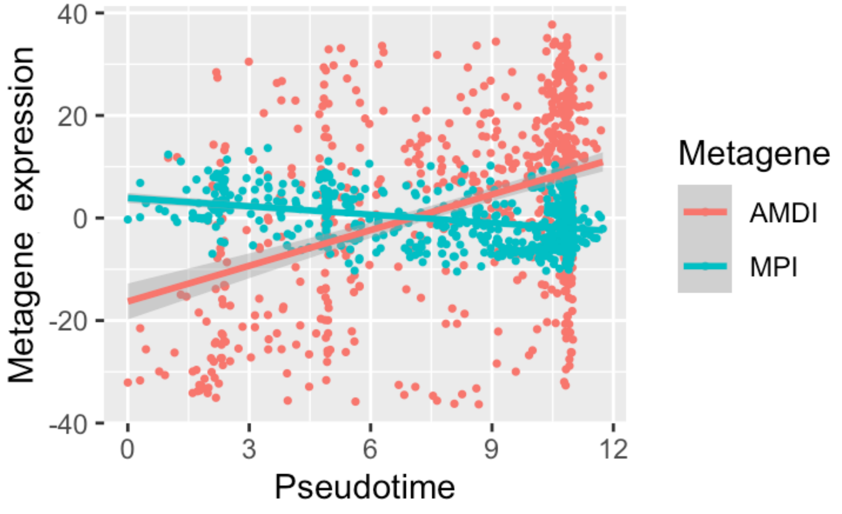

Supplemental Figure 5: Correlation of AMDI and MPI with pseudotime values in tumor core (A) and periphery (B). AMDI is positively correlated with pseudotime in both core (Pearson correlation 0.29, $p<0.0001$) and periphery (Pearson correlation 0.41, $p<0.0001$). MPI is positively correlated with pseudotime in core (Pearson correlation 0.46, $p<0.0001$) and negatively correlated with pseudotime in periphery (Pearson correlation -0.34, $p<0.0001$)

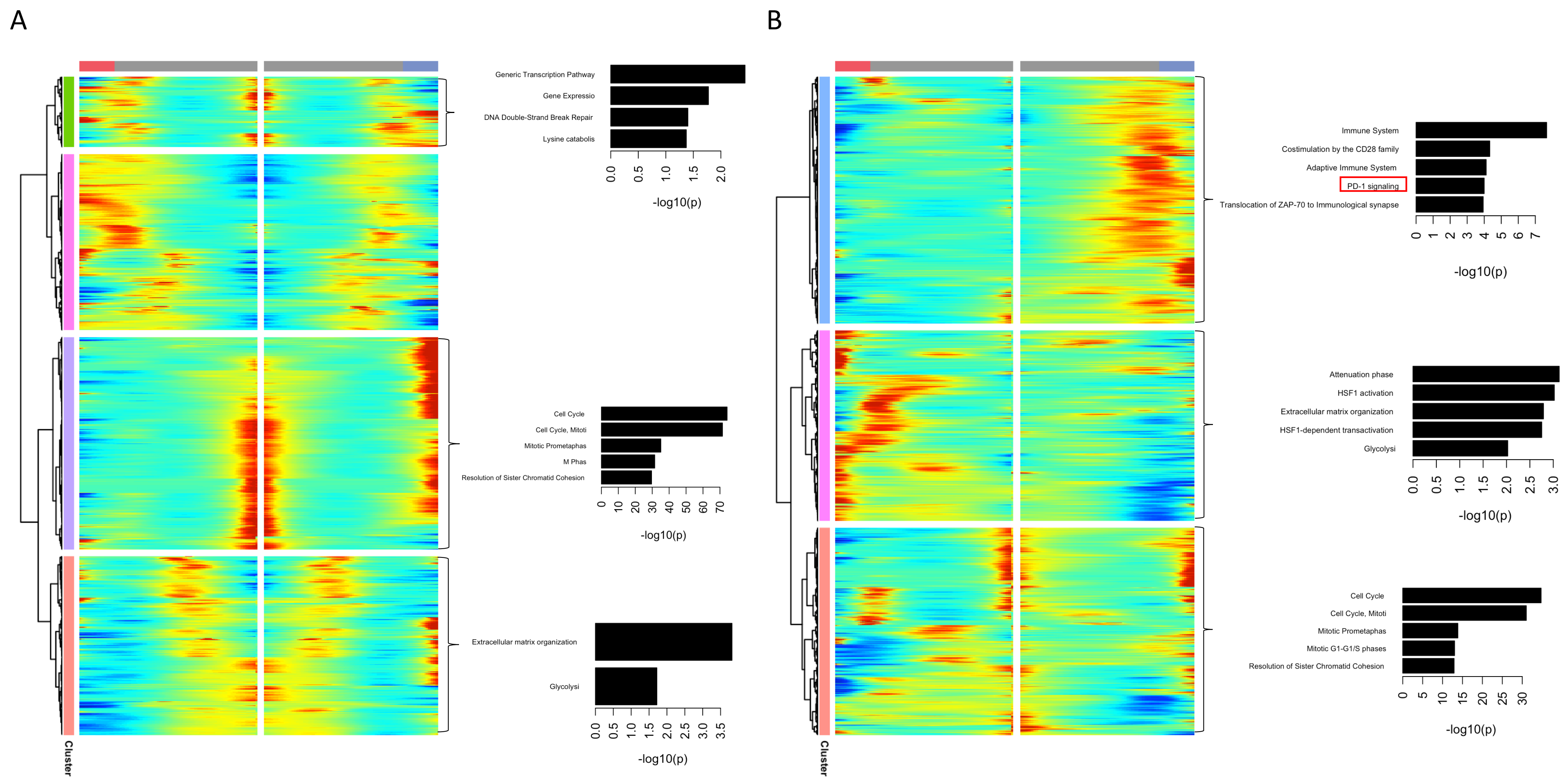

Supplemental Figure 6: Annotation of branch-specific gene clusters from tumor core. A: Annotation of branch 1 clusters with Enrichr (Reactome). Clusters as per Figure 5. Top 5 processes by Bonferroni adjusted p-values are included in barplots. Only terms with adjusted $p < 0.05$ are included. B: Annotation of branch 2 clusters.

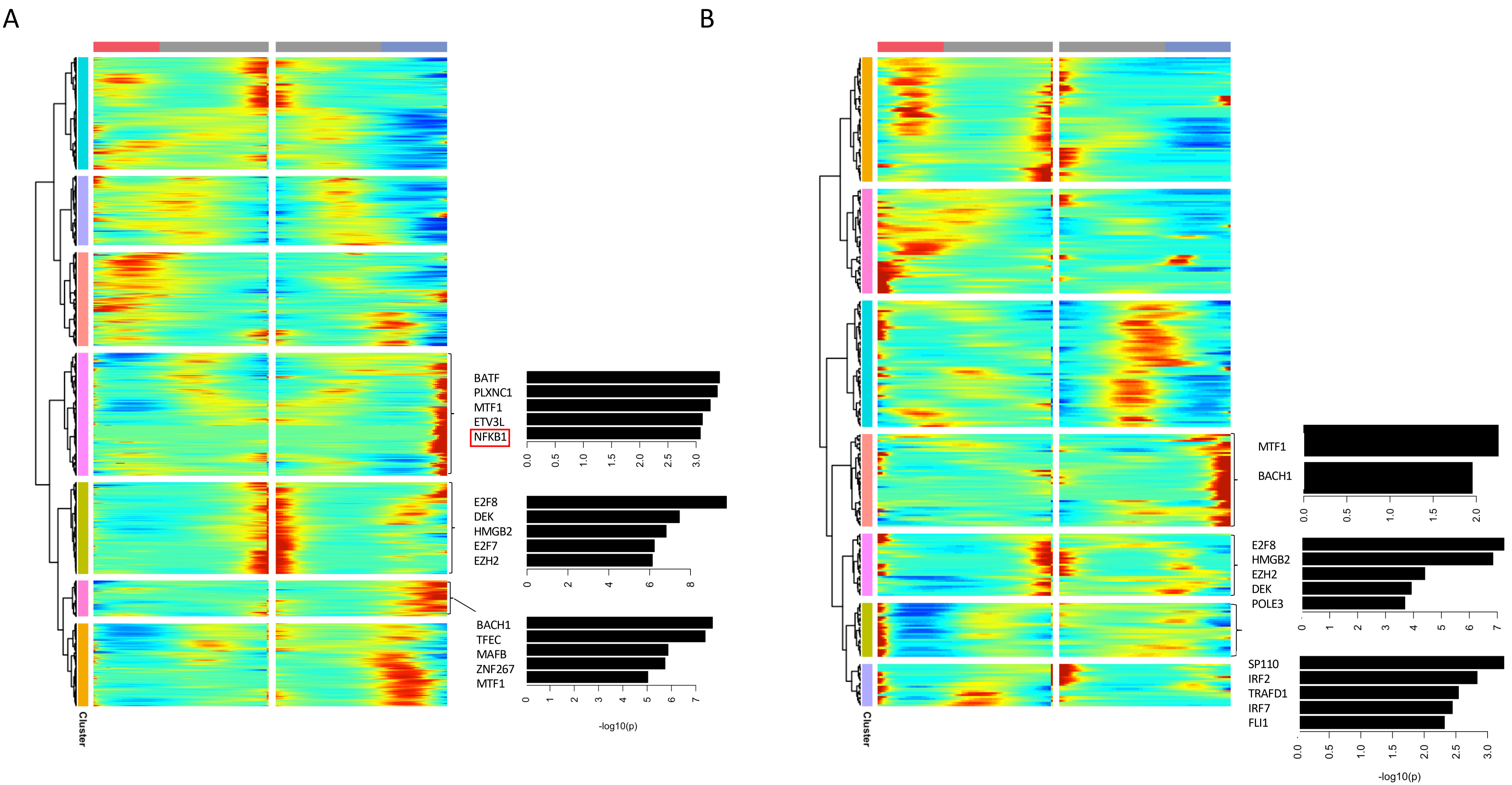

Supplemental Figure 7: Annotation of branch-specific gene clusters from tumor periphery. A: Annotation of branch 1 clusters with Enrichr (ARCHS4 transcription factors). Clusters as per Figure 6. Top 5 processes by Bonferroni adjusted p-values are included in barplots. Only terms with adjusted $p < 0.05$ are included. B: Annotation of branch 2 clusters.

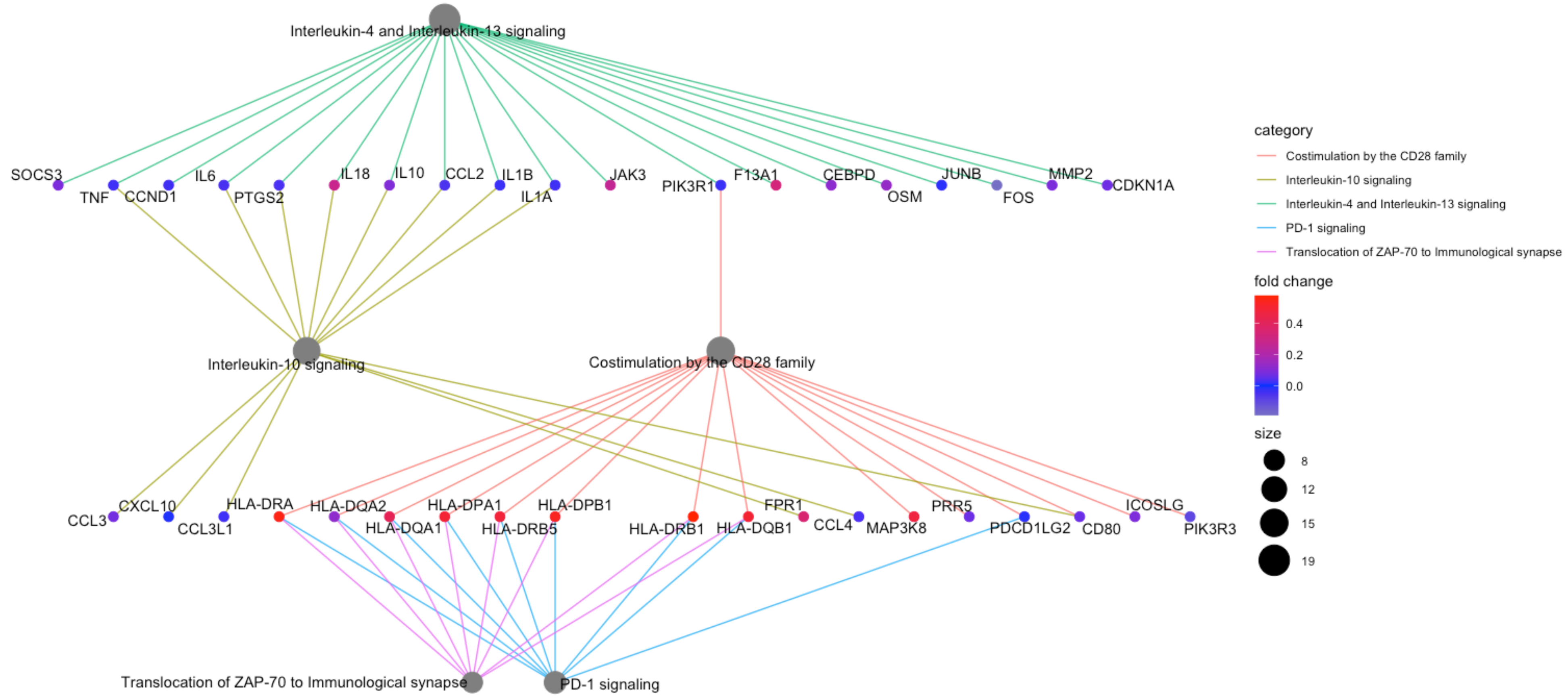

Supplemental Figure 8: Sugiyama-style Category Netplot (CNET plot) of the PD-1 associated cell population in the core tumor cell branch analysis (i.e. cluster 3 in branch 2 of tumor core cells). The CNET depicts the linkages of genes and biological concepts as a network, showing the genes that are involved in enriched pathways as well as those that belong to multiple annotation categories. The size of the GO terms are reflected by their p-values (i.e. more significant terms are larger). A p-value cutoff of 0.05 is used with Bonferroni adjustment. These gene enrichment pathways are based on the Reactome Pathway database.

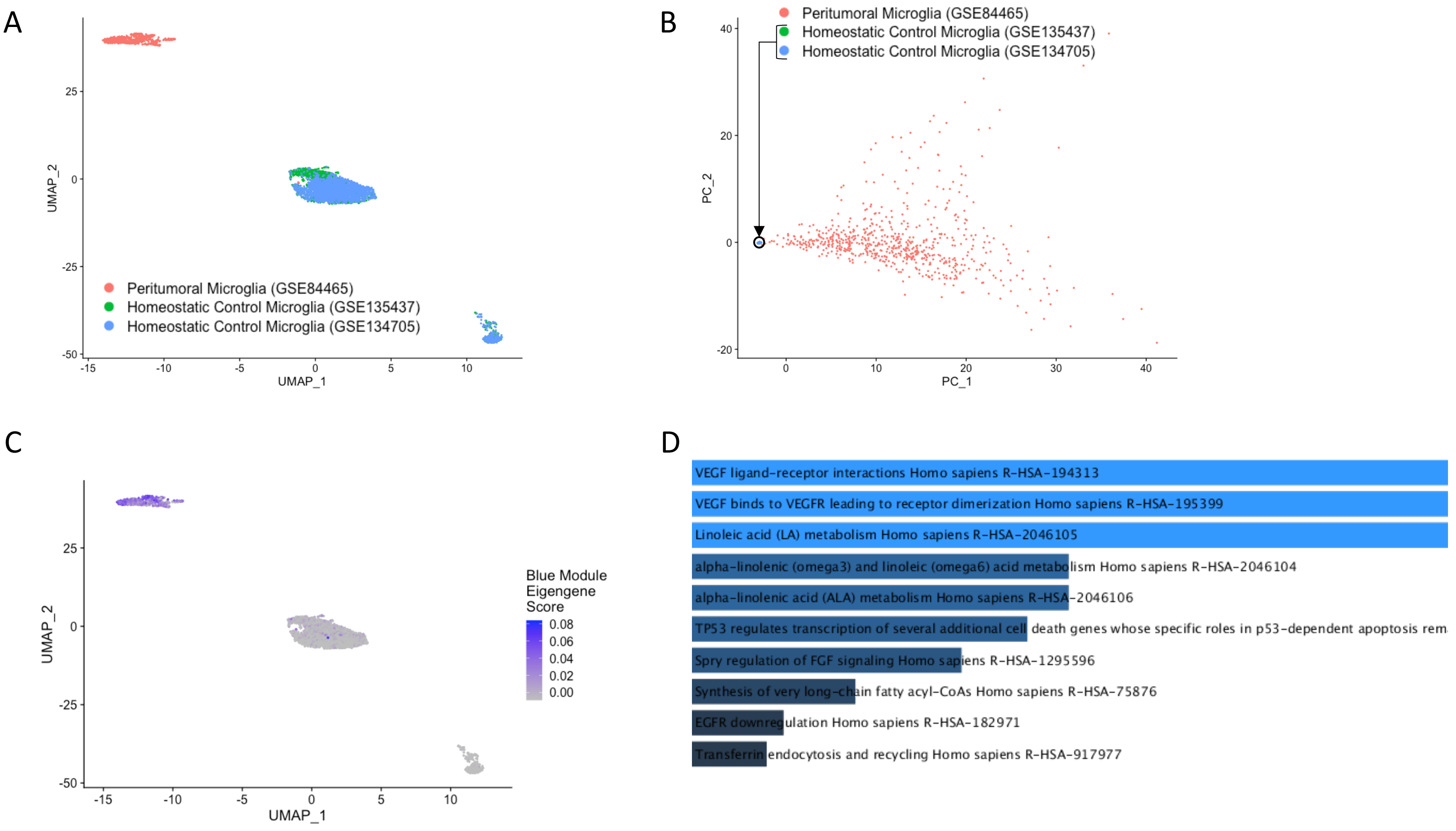

Supplemental Figure 9: Tumor vs homeostatic microglia. UMAP (**A**) and PCA (**B**) summary plots of microglia single-cell expression data from three separate studies, demonstrating that peritumoral microglia have a different expression profile compared to homeostatic microglia which show similar profiles across studies. **C:** UMAP representation colored by values of the "blue" module eigengene from WGCNA analysis, showing specificity to peritumoral microglia. **D:** Enrichr Reactome 2016 pathways of the genes in the blue module, sorted by combined score ranking ($p<0.05$).

Immune Cell	Tumor Periphery N (%)	Tumor Core N (%)
BMDM	131 (10.5)	930 (39.7)
Microglia	458 (36.8)	169 (7.2)

Supplemental Table 1: Proportion of bone marrow derived macrophages (BMDM) and microglia (as thresholded with AUCell) overall.

Cluster	N cells (% core)	BMDM N (%)	Microglia N (%)
0	303 (87.4)	248 (81.8)	45 (14.9)
1	243 (98.3)	194 (79.8)	3 (1.2)
2	242 (97.9)	1 (0.4)	0 (0.0)
3	229 (99.6)	0 (0.0)	0 (0.0)
4	225 (8.9)	0 (0.0)	0 (0.0)
5	223 (87.4)	186 (83.4)	37 (16.6)
6	215 (75.3)	0 (0.0)	0 (0.0)
7	210 (3.8)	16 (7.6)	166 (79.0)
8	203 (3.4)	8 (3.9)	169 (83.2)
9	180 (97.2)	119 (66.1)	55 (30.6)
10	178 (96.1)	0 (0.0)	0 (0.0)

11	163 (10.4)	0 (0.0)	0 (0.0)
12	142 (79.6)	57 (40.1)	3 (2.1)
13	135 (1.5)	25 (18.5)	107 (79.2)
14	130 (86.9)	0 (0.0)	0 (0.0)
15	123 (78.9)	81 (65.9)	37 (30.1)
16	117 (96.6)	51 (43.6)	2 (1.7)
17	88 (84.1)	66 (75.0)	2 (2.3)
18	80 (41.2)	0 (0.0)	0 (0.0)
19	74 (0)	0 (0.0)	0 (0.0)
20	44 (81.8)	7 (15.9)	1 (2.3)
21	42 (90.5)	2 (4.8)	0 (0.0)

Supplemental Table 2: Proportion of bone marrow derived macrophages (BMDM) and microglia (as thresholded with AUCell) by Seurat cluster.
